# Commentator Discussion: Five-year experience with titanium mesh for rigid chest wall reconstruction

**DOI:** 10.1016/j.xjtc.2024.09.011

**Published:** 2024-09-20

See Article page 180.

Presenter: Dr Sadia Tasnim

**Dr Gaetano Rocco***(New York, NY)*. Dr Tasnim, this was a very nicely presented report about the off-label use of titanium mesh on 22 patients operated in your institution since 2019. It is a fact that the ideal prosthesis for chest wall reconstruction, as we just heard from the Mayo Clinic experience, has not been found yet. And I agree with you and your colleagues that titanium is an intriguing biocompatible material. I have some questions for you. The doctor mentioned [inaudible] has championed using this same mesh since 2018 at Memorial. And since then, we've been using it off-label for selected patients undergoing redo chest wall surgery or with heavily irradiated ulcerated and infected surgical fields. It seems to me that your indications also include first-time chest wall resections and relatively limited chest wall defects. In fact, in your series, 64% of the titanium mesh reconstructions were done after removal of 3 ribs or fewer and for overall chest wall defects with an average size of 13 cm. For such defects, we as well as others may use some rigid biologic meshes or even time-honored, more conventional materials. Can you clarify your rationale to use this rather expensive titanium mesh for first-time chest wall reconstructions or when limited defects need to be covered? This is my first question.

**Figure:**
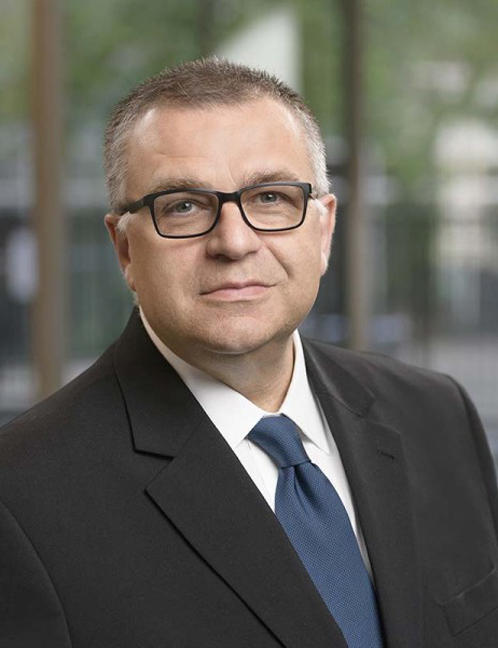

**Dr Sadia Tasnim***(Cleveland, Ohio)*. Thanks so much, Dr Rocco, for your question. In terms of size, I don't think size limited us in terms of use. We have used titanium mesh in different sizes, small or large. The largest, as I mentioned, was 495. What was the other one you said you had an indication for? Infected field?

**Figure:**
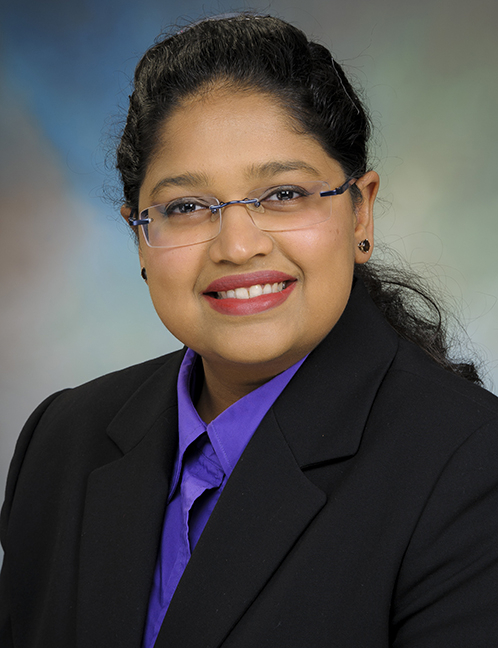

**Dr Rocco**. Yeah. Irradiated and—

**Dr Tasnim**. Ah, irradiated, yes. We use that titanium mesh for that too. Infected field, not so much due to risk of seeding the prosthesis. We do not want an infected metal prosthesis. Is there a better option to use instead of that? Biologic is an option; however, we do understand that we are compromising respiratory function when we are using nonrigid biologic material. Is there a great option? I don't know. I'm happy to have anyone who has better experience with that to come comment and we can have a discussion about it.

**Dr Rocco**. We did publish something about it. Maybe we can discuss it another time.

**Dr Tasnim**. That'll be great.

**Dr Rocco**. Titanium prosthetic material can fracture and become unstable as already demonstrated by the experience on titanium plating. We recently published our technique to anchor the titanium mesh with 3 or 4 screws. Only on 1 bony side of the chest wall defects and then loosely suture the mesh in other strategic points of the perimeter to ensure a better distribution on the mesh of the sheer force that is exerted on the mesh. Can you please describe better your actual technique to avoid fracture?

**Dr Tasnim**. Thank you so much. The one adjustment that I mentioned in my talk is that we limited the number of fixation points. Now in terms of whether we did it on one side or the other, not really. We just used fewer points of fixation as we went along in our experience. And the reason we did that is we believe that the mesh gets incorporated soon enough and even though it fractures, it does not migrate and so far, we have not had any mesh, or a part of mesh herniate into any organ or through the skin.

**Dr Rocco**. The last question. Firstly, I didn't have your slides, but I had your paper, thank you very much. And there were 5 instances of local wound infection, and in 2 cases I read, the mesh was actually involved and had to be removed. Now, according to the literature, the use of polytetrafluoroethylene or methyl methacrylate has been associated with local infection and prosthesis removal rates <10%. Recent data showed even better results with biological prostheses. What would be your strategy to avoid local wound infection? Can a predisposing factor be the combination of a titanium prosthesis with a nonreabsorbable Prolene mesh like you have been using? Or would a more liberal use, like I said before, of free flaps contribute to better tissue coverage, thereby reducing the local infection complication rate?

**Dr Tasnim**. Thank you so much. I apologize for not sending you the slides earlier. Hopefully, I have not deviated from my manuscript too much. In terms of reducing infections, first, second, and third mesh coverage, mesh coverage, mesh and coverage. We use that with free flap or rotational flap, mostly a rotational flaps, less free flaps, due to challenges we already know. We do encourage our plastic colleagues to take the drains out, so it becomes one less nidus for infection. Antibiotic coverage. We can extend our antibiotic coverage if we believe it's clinically indicated. Otherwise, I don't think there is any other particular special consideration that we take. Additionally, the infections reported are over the lifetime of the mesh which spans up to five years. The numbers you cite for PTFE mesh are for perioperative infection rates and they increase significantly over the lifetime of the mesh. We, notably, did not have a single perioperative infection requiring mesh revision in this study. Thank you.

**Dr Daniel P. Raymond**. Just a few points and details. Sadia did a wonderful job presenting. She's actually not done one of these operations. She's participated in video, so some of the technical stuff is challenging for her. I think the defect size issue, you're just seeing an evolution in practice, and now I really only use titanium mesh for larger defects, more in the 4 or 5 rib area. It was a new material that we were trying to determine how to fit into our practice thus the smaller defects earlier on in the experience. That being said, a three rib anterior defect certainly warrants rigid repair in our opinion. As far as cost goes, it's the same as polytetrafluoroethylene, for us at least. There's no difference in cost. So, I guess we get a good deal or something. I don't know. But it's not an expensive material, frankly.

**Unidentified Speaker 2**. Thanks. Nice talk. Question for you. Have you guys tried using a titanium neorib system by MedXpert (MedXpert GmbH)? I think it would give you some of the benefits of titanium. It's off the shelf, I think similar cost. I think it might be a little less likely to fracture. I think the only disadvantage is getting into the sternum can be tricky with that product. Have you seen that or used it, and why do you use this instead?

**Dr Tasnim**. Enlighten me on the neorib system or these rib plates you're talking about.

**Unidentified Speaker 2**. Yeah. It's a rib reconstruction system made by—it's called Stratos or Stractos. I think it was originally from Europe.

**Dr Raymond**. There is 1 that is approved for gap defects. Read the paperwork on it, because what they say is this is not a permanent solution to the problem. You need to take it out. And how many of us go back in and take out our chest wall reconstructions? And then they also say that these devices are not meant to replace ribs. And that's, I think, a global message that everyone needs to understand. Rib plating is not new ribs. They cannot replace ribs. They are cleared for less than 2-cm gaps, and they will break. They are unstable. They are not meant for that kind of force. And the package insert from the manufacturer says the same thing.

**Unidentified Speaker 2**. That's good to know. I haven't had issues myself with [crosstalk]—

**Unidentified Speaker 3**. While you were up there, there was a comment made about the fixation and the technicalities of how much to fix and where to fix. Would you like to comment on that?

**Dr Raymond**. The discussion about the gasket is because it's the sharp edges of the mesh that tear your gloves apart. And so, you don't want to break sterility—so the gasket protects that and keeps you from having breaks in technique. The mesh, when you get it in and if you don't have that gasket, you can't move it. It just sticks to everything and it's hard to move around and position. And so that's why I use the gasket, it permits mesh adjustment. Regarding the fixation, once we figured that out, we just anchored along 1 rib. And so technically what I do now is usually 3 screws along 1 rib. The bigger issue is when you're doing really large lateral chest wall resections, you need to aggressively bend and shape the plate because it will have a memory and can informally pop back out. I've had patients where it's kind of popping out in their axilla. I hadn't had to go in and revise them, but it's just not satisfying to look at or how you're imaging them.

**Dr Rocco**. One more comment. The same fibrotic reaction that GoreTex (W. L. Gore & Associates) evokes in the tissue, is what we have to use for our prosthetic materials, especially with titanium because after 3 weeks it's going to be incorporated. That's 1 thing. The second thing I forgot to ask you. Our experience in the beginning with this mesh was that we had a pleural effusion forever. Because the friction of the visceral pleura against the mesh would create this kind of scenario. So, we added a biological, usually an inexpensive mesh as a first layer, and then on top of that, the titanium mesh. Did you find in your experience this?

**Dr Tasnim**. We've only found 1 pleural effusion, and it was not clinically that significant.

**Unidentified Speaker 3**. Yeah, actually I think the fibrosis is a good thing, and that's why we don't see a hernia recurrence. Any incorporated meshes when you see that [inaudible]. That was something I looked at; I was like “We never see that. I've never seen a hernia [inaudible].”

**Dr Tasnim**. Thank you.

## Conflict of Interest Statement

Dr Raymond is a development partner and invited speaker for KLS Martin and holds an equity interest in Zimmer). All other authors reported no conflicts of interest.

The *Journal* policy requires editors and reviewers to disclose conflicts of interest and to decline handling or reviewing manuscripts for which they may have a conflict of interest. The editors and reviewers of this article have no conflicts of interest.

